# Novel Type II and Monomeric NAD^+^ Specific Isocitrate Dehydrogenases: Phylogenetic Affinity, Enzymatic Characterization, and Evolutionary Implication

**DOI:** 10.1038/srep09150

**Published:** 2015-03-16

**Authors:** Peng Wang, Changqi Lv, Guoping Zhu

**Affiliations:** 1 Institute of Molecular Biology and Biotechnology, College of Life Sciences, Anhui Normal University, No.1 Beijing East Road, Wuhu 241000, Anhui, China

## Abstract

NAD^+^ use is an ancestral trait of isocitrate dehydrogenase (IDH), and the NADP^+^ phenotype arose through evolution as an ancient adaptation event. However, no NAD^+^-specific IDHs have been found among type II IDHs and monomeric IDHs. In this study, novel type II homodimeric NAD-IDHs from *Ostreococcus lucimarinus* CCE9901 IDH (OlIDH) and *Micromonas* sp. RCC299 (MiIDH), and novel monomeric NAD-IDHs from *Campylobacter* sp. FOBRC14 IDH (CaIDH) and *Campylobacter curvus* (CcIDH) were reported for the first time. The homodimeric OlIDH and monomeric CaIDH were determined by size exclusion chromatography and MALDI-TOF/TOF mass spectrometry. All the four IDHs were demonstrated to be NAD^+^-specific, since OlIDH, MiIDH, CaIDH and CcIDH displayed 99-fold, 224-fold, 61-fold and 37-fold preferences for NAD^+^ over NADP^+^, respectively. The putative coenzyme discriminating amino acids (Asp326/Met327 in OlIDH, Leu584/Asp595 in CaIDH) were evaluated, and the coenzyme specificities of the two mutants, OlIDH R^326^H^327^ and CaIDH H^584^R^595^, were completely reversed from NAD^+^ to NADP^+^. The detailed biochemical properties, including optimal reaction pH and temperature, thermostability, and metal ion effects, of OlIDH and CaIDH were further investigated. The evolutionary connections among OlIDH, CaIDH, and all the other forms of IDHs were described and discussed thoroughly.

The progressive sequencing of complete biological genomes has dramatically increased the size of protein databases. The expansion of protein information, most of which are functionally annotated by computational techniques, will consequently increase the diversity of each protein family, thus providing us with an opportunity to extend and refine the classification of protein families. Protein phylogenetic analysis has always been important, because it can provide insight into protein evolution and further implicate protein function. In the present study, we applied this principle to explore novel isocitrate dehydrogenases (IDHs).

IDH is a key enzyme in the tricarboxylic acid (TCA) cycle. It catalyzes the oxidative decarboxylation of isocitrate to α-ketoglutarate (α-KG) and CO_2_, which is accompanied by the reduction of NAD(P^+^) to NAD(P)H. The IDH reaction provides organisms with not only energy but also biosynthetic precursors, such as α-KG, for metabolism. Thus, these metabolic pathways are among the first to have evolved^1^,^2^. Consequently, IDHs are ubiquitously distributed throughout the three domains of life: Archaea, Bacteria, and Eukarya. Based on coenzyme specificity, the IDH family can be divided into NAD^+^-dependent IDHs (EC 1.1.1.41, NAD-IDHs) and NADP^+^-dependent IDHs (EC 1.1.1.41, NADP-IDHs). IDHs with different coenzyme dependencies play varying roles *in vivo*. NAD-IDH catalysis generates NADH, which participates in energy metabolism. NADP-IDH catalysis generates NADPH, which is an important source of reducing power. NADPH also plays a role in the cellular defense against oxidative damage and the detoxification of reactive oxygen species^3^,^4^,^5^.

Most studies have focused on the general understanding of the biochemistry, structure, and evolution of IDH. Meanwhile, the potential applications of IDH in biotechnology and drug design against pathogens have also been recently investigated. An NAD-IDH with poor performance in decarboxylating from *Zymomonas mobilis* serves as a potential genetic modification target towards optimized *Z*. *mobilis* strains to produce ethanol^6^. The characterization of NADP-IDH from *Microcystis aeruginosa* may provide new ideas for controlling blue-green algae through biological techniques^7^. IDHs from pathogenic bacteria, such as *Plasmodium falciparum*, *Mycobacteriu tuberculosis*, and *Leptospira interrogans*, have been reported as drug targets, because they stand at a branch point of the TCA cycle and glyoxylate shunt^8^,^9^,^10^. IDHs are also ideal immuno-diagnostic candidates, due to their highly conserved housekeeping function. For example, *M. tuberculosis* IDHs elicit strong B-cell responses in tuberculosis (TB)-infected populations and can differentiate between healthy vaccinated and TB populations^11^. In addition, *Helicobacter pylori* IDH can be an immunogen that interacts with the host immune system to subsequently lead to possible autolytic release and significantly elicit humoral responses in individuals with invasive *H. pylori* infection^12^.

Besides pathogenic bacterial IDH, human cytosolic NADP-IDH (IDH1) and mitochondrial NADP-IDH (IDH2) have been considered as drug targets. Mutations in IDH1 and IDH2 are frequently identified in various cancers, such as glioblastoma multiforme and acute myeloid leukemia^13^,^14^. Heterozygous IDH mutations are remarkably specific to a single codon in the conserved and functionally important arginine 132 residue (R132) of IDH1 and 172 residue (R172) of IDH2. Mutations result in the simultaneous loss of normal IDH catalytic activity. However, the production of α-KG and NADPH grants mutated IDHs with the neomorphic activity of reducing α-KG to 2-hydroxyglutarate (2-HG), which is accompanied by the oxidation of NADPH to NADP^+^ 15,16. The accumulation of 2-HG competitively inhibits α-KG-dependent enzymes, thus causing cellular alterations in epigenetics, collagen maturation, and hypoxia signaling^17^,^18^,^19^.

As an ancient enzyme, IDH acquired various primary structures and different oligomeric states through evolution. Four kinds of IDHs have been reported: monomer, homo-dimer, homo-tetramer, and hetero-oligomer. Monomeric IDHs have been characterized from various eubacteria, and all of them are highly specific to NADP^+^ 20,21,22. Because the amino acid sequence identities are <10% between monomeric IDHs and other types of IDHs, this group has been recognized as a separate clade that evolved independently^20^,^23^. Dimeric and multimeric IDHs have been divided into three phylogenetic subfamilies^23^,^24^,^25^. Subfamily I is a prokaryotic group, in which NAD^+^ and NADP^+^ usage is widespread within archaeal and eubacterial homo-dimeric enzymes. Subfamily II is mainly composed of eukaryotic homo-dimeric NADP-IDHs, with a small number of eubacterial homo-dimeric NADP-IDHs. Subfamily III is comprised of mitochondrial hetero-oligomeric NAD-IDHs and eubacterial homo-tetrameric enzymes with either NAD^+^ or NADP^+^ as the cofactor^26^.

Because subfamily III IDHs share more than 30% sequence identities with subfamily I members but less than 15% sequence identities with subfamily II counterparts, subfamilies III and I can be combined accordingly. Therefore, we simply divided the IDH protein family into type I IDHs (subfamily I and III) and type II IDHs (subfamily II) in our previous study^4^. Three total types of IDH can be distinguished: type I IDH, type II IDH, and monomeric IDH. Interestingly, both NAD^+^ and NADP^+^ are broadly utilized among type I IDHs. However, homo-dimeric type II IDHs and monomeric IDHs are all NADP^+^ specific^4^. We previously demonstrated that NAD^+^ use is an ancestral trait of IDH, and the NADP^+^ phenotype of eubacterial dimeric NADP-IDH arose on or about the time that eukaryotic mitochondria first appeared (about 3.5 billion years ago) to synthesize NADPH for the adaptation of bacterial growth on acetate^4^. Consequently, NADP^+^-specific type II IDHs and monomeric IDHs may also have their own NAD^+^-specific ancestors. However, no NAD-IDHs that belong to these two subfamilies have been explored.

The rapid growth of the IDH protein “pool”, which has been contributed by various genome projects, should allow us to search for putative NAD^+^-specific type II IDHs and monomeric IDHs. In the present study, four novel NAD-IDHs that belong to the families of type II IDHs and monomeric IDHs were reported for the first time. Enzymatic properties of these NAD-IDHs, including the oligomeric state in solution, optimum pH and temperature for catalysis, thermostability, and metal ion dependency, were characterized thoroughly. Kinetic parameters of the two IDHs towards coenzymes were determined in detail, and their coenzyme-binding sites were evaluated by site-directed mutagenesis. The discovery of these novel NAD-IDHs will refine the chemistry and phylogeny of IDH and provide new insights into the evolution of this ancient protein family.

## Results

### Phylogenetic Analysis and Sequence Alignment

**Figure 1:**
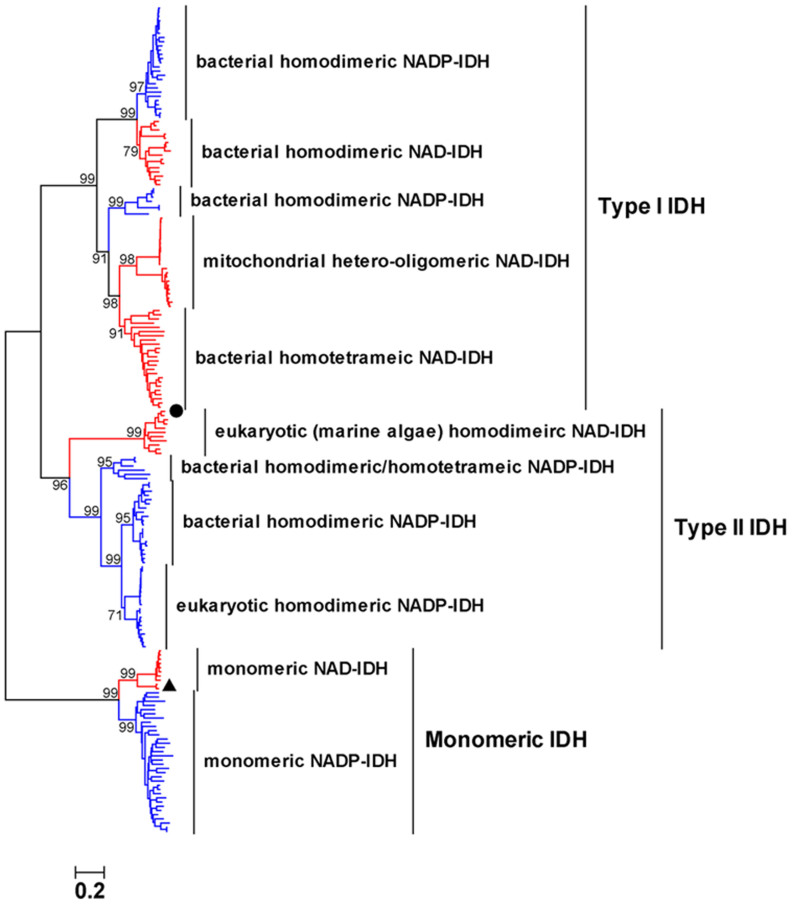
Evolutionary relationships of 197 IDHs from diverse background. The evolutionary history was inferred using the Neighbor-Joining method. The percentage of replicate trees in which the associated taxa clustered together in the bootstrap test (500 replicates) are shown next to the branches. The tree is drawn to scale, with branch lengths in the same units as those of the evolutionary distances used to infer the phylogenetic tree. Phylogenetic analyses were conducted in MEGA6. The IDH sequences used were listed in Table S1. OlIDH and CaIDH were marked by “
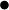
” and “
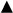
” respectively.

In our previous study, we divided the IDH protein family into type I IDHs and type II IDHs based on sequence homology^4^. Herein, we expanded the classification of this ancient family by incorporating new members (Fig. 1). Monomeric IDHs were included, although their overall sequence homologies with other forms of IDH are relatively low (≈10%)^22^,^27^. Consequently, they constituted a monophylogenic clade on the phylogenetic tree (Fig. 1). Therefore, three main IDH subfamilies (type I IDH, type II IDH and monomeric IDH) were identified to comprise the whole IDH protein family. Members in IDH subfamily were further classified into eleven subgroups with regard to different coenzyme specificity, different oligomeric states and diverse resources (Fig. 1). Different tree building algorithms were also employed, such as UPGMA method, Maximum Likelihood method and Minimum Evolution method. All trees contain three well-supported monophyletic groups: type I IDHs, type II IDHs, and monomeric IDHs (see Supplementary Fig. S3, Fig. S4 and Fig. S5 online). When an outgroup of malate dehydrogenase (MDH) was added into the phylogenetic study, the overall topology of the evolutionary tree remained very similar (see Supplementary Fig. S6 online).

In the type I subgroup, the most common ones are the eubacterial homodimeric NADP- and NAD-IDHs, which are represented by *E. coli* IDH and *Z. mobilis* IDH, respectively^6^,^23^. The pairwise amino acid sequence identity among this subgroup was more than 50%. Mitochondrial hetero-oligomeric NAD-IDHs, together with a batch of eubacterial homotetrameric NAD-IDHs, were grouped into a single branch in the type I subfamily. More than 45% identity exists between these eukaryotic and prokaryotic NAD-IDHs^26^. Although this branch was designated as subfamily III in other IDH phylogenetic studies^20^,^24^,^25^,^28^, it is reasonable to encompass it into the type I subfamily, because proteins in this branch exhibit more than 30% identity with type I eubacterial homodimeric IDHs. A small group of IDHs, which were represented by *Rickettsia* IDH and *Thermus thermophilus* IDH, was branched before the mitochondrial group. These IDHs were structurally distinguished from other dimeric IDHs, as they were longer in the C-terminal region. Although they were assigned as subfamily IV IDHs in two recent studies^28^,^29^, they were included as type I IDHs in our study, as they shared considerable sequence identities (>40%) with mitochondrial NAD-IDHs.

The type II subfamily was comprised of homodimeric NADP-IDHs from eukaryotes and eubacteria (Fig. 1). A small group of NADP-IDHs, which are represented by *Thermotoga maritima* IDH (TmIDH)^30^, branched clearly before the well-characterized homodimeric NADP-IDH clade. Although the TmIDH-like proteins were highly identical (>50%) to type II homodimeric NADP-IDHs, these two kinds of IDHs branched separately in the type II subtree. This separate branching may be due to the fact that TmIDH-like IDHs can form homotetramers in solution under some conditions^25^,^30^. Because all reported type II IDHs have been NADP^+^ specific, no NAD-IDHs have been found in this subfamily. In this study, we identified the type II NAD-IDH group for the first time. This small group of distinctive IDHs was represented by IDH from *O*. *lucimarinus* CCE9901 (OlIDH), along with several counterparts that are derived from marine algae, such as *Micromonas* sp. RCC299 (MiIDH, GenBank accession number: XP_002502450). OlIDH demonstrated substantial sequence identity (>30%) with typical type II homodimeric NADP-IDHs and low identity with type I IDHs (<15%). Therefore, OlIDH clustered into a unique clade among the type II subfamily.

**Figure 2:**
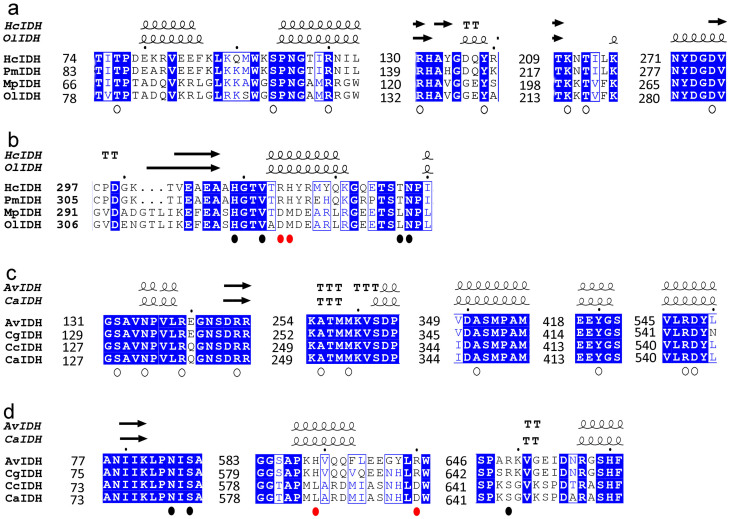
Secondary structure based protein sequence alignments. (a, b), The comparisonof OlIDH with its homologous IDH from *Micromonas pusilla*(MpIDH, GenBank Accession No. XP_003056989.1) and two typical type II homodimeric NADP-IDHs from human cytosol (HcIDH, GenBank Accession No. NP_001269316.1) and porcine mitochondria (PmIDH, GenBank Accession No. P33198.1). (c, d), The comparisonof CaIDH with its homologous IDH from *Campylobacter curvus*(CcIDH, GenBank Accession No. WP_018136314.1) and two typical monomeric NADP-IDHs from *Azotobacter vinelandii* (AvIDH, GenBank Accession No. BAA11169.1) and *Corynebacterium glutamicum* (CgIDH, GenBank Accession No. WP_011013800.1). High-resolution structure of HcIDH (PDB ID, 1T09) and AvIDH (PDB ID, 1J1W) were downloaded from the PDB database. The homology models of OlIDH and CaIDH were generated by SWISS-MODEL server (http://swissmodel.expasy.org/). Invariant residues are highlighted by shaded blue boxes and conserved residues by open blue boxes. The conserved residues involved in the substrate binding are indicated by empty circle (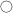). The core cofactor binding sites, which are the targets of the site directed mutagenesis, are indicated by red dots (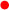) while other cofactor binding pocket residues are indicated by black dots (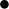). The figure was made with ESPript 2.2.

Although OlIDH was recognized as a member of the type II subfamily, its coenzyme specificities seemed to be different from those of the other type II NADP-IDHs. The crystal structure of human cytosolic homodimeric NADP-IDH, which is the most investigated type II IDH, shows that Arg353 and His354 were involved directly in coenzyme discrimination^31^. The conservation of these two NADP^+^-binding residues was confirmed by the structures of type II NADP-IDHs from *M. tuberculosis*^9^, *Desulfotalea psychrophila*^32^, *Thermotoga maritima*^30^, porcine mitochondria^33^, and yeast mitochondria^34^. However, the corresponding amino acid residues in OlIDH were substituted with Asp326 and Met327 (Fig. 2). The replacement of two positively charged amino acids with one negatively charged amino acid (Asp) and one neutral amino acid with a large side chain (Met) suggests that OlIDH should favor its binding to NAD^+^ over NADP^+^, because Asp in the coenzyme binding site will repel the 2'-phosphate of NADP^+^ but can properly contact with NAD^+^ 35,36. Other suspected type II NAD-IDHs that were grouped with OlIDH also had Asp and Met (or Leu) in the corresponding sites (see Supplementary Fig. S1 online), suggesting that they share the same coenzyme-binding mechanism.

All monomeric IDHs were separated into a monophyletic group in the phylogenetic tree (Fig. 1). Two subgroups could be distinguished in the clade clearly, one of which was represented by the well-studied monomeric NADP-IDHs from *Azotobacter vinelandii*(AvIDH)^21^ and *Corynebacterium glutamicum* (CgIDH)^20^. The other, however, was newly discovered and was proposed to be NAD^+^ specific. The representative member of this special subgroup was the *Campylobacter* sp. FOBRC14 IDH (CaIDH). CaIDH was 731 amino acids in length, which is typical for monomeric IDH. However, CaIDH shared less than 50% sequence identity with monomeric NADP-IDHs, whereas monomeric NADP-IDHs shared more than 70% sequence identity among themselves. Sequence alignment results show that the key coenzyme-binding residues, His589 and Arg600, in AvIDH^20^, which are absolutely conserved in all monomeric NADP-IDHs, had been replaced by Leu and Asp at the corresponding sites of CaIDH (Fig. 2). The presence of negatively charged Asp and neutral Leu with a large side chain eliminates the possibility of NADP^+^ use by CaIDH, thus making CaIDH the first putative NAD^+^-specific monomeric IDH. Besides CaIDH, some other homogeneous monomeric NAD-IDHs, which are derived from *Campylobacter* species, such as *Campylobacter curvus* (CcIDH, GenBank accession number: WP_018136314), were also found (see Supplementary Fig. S2 online). These characteristic IDHs shared more than 70% sequence identity with each other, and they all had Leu and Asp (or Leu) at the putative coenzyme-binding sites (see Supplementary Fig. S2 online).

### Overexpression, Purification, and Oligomeric State Determination

**Figure 3:**
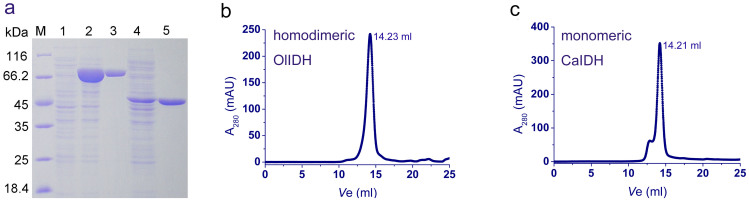
Overexpression, purification and oligomeric state determination of the recombinant CaIDH and OlIDH. (a), Protein purity was detected by 12% SDS-PAGE. M, protein marker; lane 1, crude extracts of cells harboring pET-28b(+) with IPTG induction; lane 2, crude extracts of cells harboring recombinant plasmid pET-*CaIDH* with IPTG induction; lane 3, purified recombinant CaIDH; lane 4, crude extracts of cells harboring recombinant plasmid pET-*OlIDH* with IPTG induction; lane 5, purified recombinant OlIDH. (b) and (c), SEC analysis of the recombinant OlIDH and CaIDH. The flow rate was 0.5 ml.min^−1^ and the proteins in the fractions were monitored at 280 nm. *V*_e_ of the recombinant OlIDH and CaIDH were 14.23 ml and 14.21 ml, respectively.

The recombinant OlIDH and CaIDH that were tagged with 6×His were successfully produced in *E. coli* and then purified to homogeneity (Fig. 3a). Purified OlIDH and CaIDH gave a single band around 45 kDa and 80 kDa in SDS-PAGE, respectively, which compared well with the theoretical molecular mass of 6×His-tagged OlIDH (46 kDa) and CaIDH (81 kDa). Size exclusion chromatography (SEC) was then performed to estimate the oligomerization status of OlIDH and CaIDH in solution. A single-elution peak was observed for OlIDH, and its native molecular mass was estimated to be 76 kDa (Fig. 3b). This data can be interpreted as the protein being presented as a homodimer in solution, since the calculated mass of a monomer is 46 kDa. As for CaIDH, a dominant peak, corresponding to a molecular weight of 81 kDa, was eluted (Fig. 3c). This proved that the overwhelming majority of CaIDH presented as a monomer in solution. The fraction (<5%) that eluted before the main peak was calculated to be 150 kDa, and this represented a dimeric CaIDH. The dimeric CaIDH was more likely to be induced by factors, such as protein concentration, salt, temperature, and pH.

**Figure 4:**
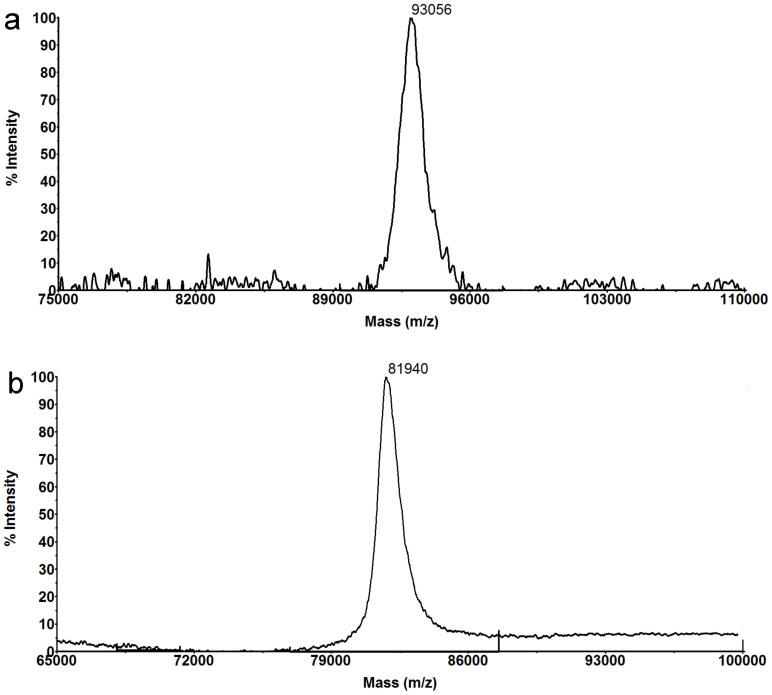
MALDI-TOF/TOF MS spectra of the recombinant OlIDH (a) and CaIDH (b).

However, molecular weight estimation by SEC is limited by the premise that the protein interacts with the column resin in an ideal way (e.g., no electrostatic or hydrophobic interactions)^37^,^38^. In our experiment, the disagreement between the SEC estimation (76 kDa) and the deduced molecular weight of the dimeric OlIDH (92 kDa) may be due to non-ideal interactions between the protein and the SEC media. In order to accurately assign the molecular masses of the recombinant OlIDH and CaIDH, we performed the MALDI-TOF/TOF mass spectrometry. The molecular weight of the recombinant OlIDH and CaIDH were precisely determined to be 93 kDa and 82 kDa, respectively (Fig. 4), demonstrating the homodimeric structure of OlIDH and the monomeric structure of CaIDH.

### Enzyme Activity and Kinetic Characterization

The specific activity of purified recombinant OlIDH was 72.3 U/mg with NAD^+^ and only 3.8 U/mg with NADP^+^. Recombinant CaIDH showed similarly high specific activity with NAD^+^ (53.2 U/mg) and very low activity with NADP^+^ (8.7 U/mg). This observation primarily confirmed the NAD^+^ preference of OlIDH and CaIDH, as suggested by the bioinformatic analysis. Kinetic characterization results show that the *K*_m_ of OlIDH for NADP^+^ was over 16-fold greater than the *K*_m_ for NAD^+^. The coenzyme specificity (*k*_cat_/*K*_m_) of OlIDH was 99-fold greater for NAD^+^ than NADP^+^ (Table 1). Consequently, OlIDH showed a high preference for NAD^+^, thus becoming the first NAD^+^-specific IDH in the type II subfamily. As expected, CaIDH was also characterized as an NAD^+^-specific IDH by kinetic analysis, and its coenzyme specificity was 61-fold greater towards NAD^+^ than NADP^+^ (Table 1). Hence, CaIDH represents the first known monomeric NAD-IDH.

Because the newly defined type II NAD-IDHs and monomeric NAD-IDHs stand for important IDH subfamilies, identification of one enzyme for each is not sufficient enough to support their distinctiveness. We therefore characterized another two NAD-IDHs that belong to these two novel subfamilies, respectively. MiIDH, an OlIDH analog from *Micromonas* sp. RCC299, and CcIDH, a CaIDH analog from *Campylobacter curvus,* were also produced in *E. coli* and purified to homogeneity. Kinetic analysis showed that both MiIDH and CcIDH are NAD^+^-specific as expected, because their preference for NAD^+^ was 224-fold and 37-fold over NADP^+^, respectively (Table 1). The conformation of the other two NAD^+^-specific members from type II IDH and monomeric IDH subfamilies further validated the novelty of our finding.

### Coenzyme Binding Site

**Figure 5:**
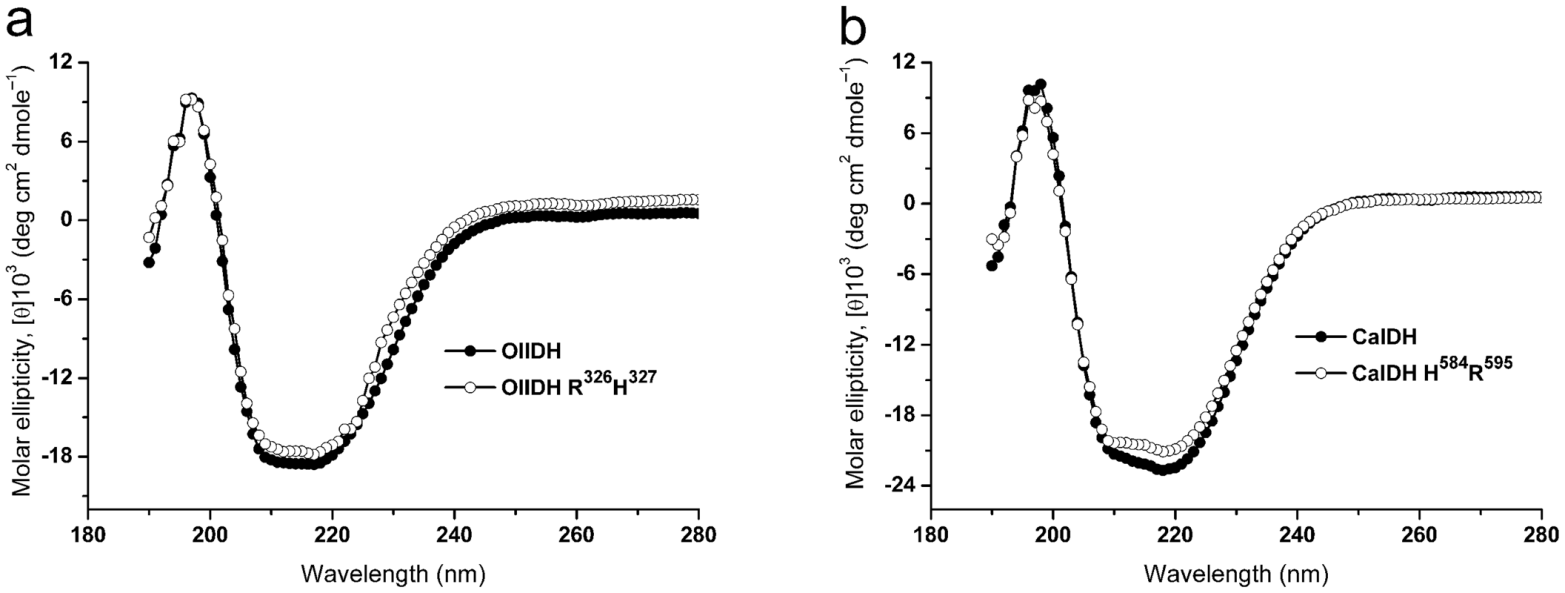
Circular diochroism (CD) spectra of the wild-type OlIDH, CaIDH and their mutants, OlIDH R^326^H^327^ and CaIDH H^584^R^595^. The CD was measured and the molar ellipticity was calculated as described in Methods section.

To evaluate the significance of the putative coenzyme-determining sites (Asp326 and Met327 in OlIDH, Leu584 and Asp595 in CaIDH), each mutant enzyme containing two point mutations, R^326^H^327^ for OlIDH and H^584^R^595^ for CaIDH, was constructed, based on the protein sequence alignment (Fig. 2). The mutated enzymes were successfully produced in *E. coli* and purified to homogeneity. CD spectroscopy was performed to determine the secondary structure of wild-type and mutant enzymes (Fig. 5). The results show that OlIDH R^326^H^327^ and CaIDH H^584^R^595^ mutants were very similar to the wild-type enzyme, thus indicating that mutations that occur at key activity sites do not cause significant changes in protein secondary structure.

The kinetic characterization results are reported in Table 1. The OlIDH R^326^H^327^ mutant displayeda 22-fold higher *K*_m_ value for NAD^+^ than that of the wild-type enzyme. Meanwhile, the mutant enzyme showed a greatly increased affinity to NADP^+^, as demonstrated by a 270-fold decrease in *K*_m_ value. The *k*_cat_/*K*_m_ of OlIDH R^326^H^327^ towards NADP^+^ was 49-fold higher than that of the wild-type enzyme, whereas the *k*_cat_/*K*_m_ for NAD^+^ underwent a 44-fold decrease. Consequently, the overall specificity of the OlIDH R^326^H^327^ mutant was 22-fold greater for NADP^+^ than that for NAD^+^. Therefore, the two point mutations in OlIDH completely altered its coenzyme specificity, which demonstrates that Asp326 and Met327 were key specificity determinants for OlIDH.

The importance of Leu584 and Asp595 in the direct binding of NAD^+^ to CaIDH was also confirmed by the mutagenesis study. The CaIDH H^584^R^595^ mutant's affinity to NAD^+^ was loosened, as evidenced by a17-fold elevation in *K*_m_, as compared to that of the wild-type enzyme. By contrast, the mutant enzyme displayed a 45-fold decrease in *K*_m_ for NADP^+^. The *k*_cat_/*K*_m_ of CaIDH H^584^R^595^ towards NADP^+^ was 92-fold higher than that of the wild-type enzyme, whereas the *k*_cat_/*K*_m_ for NAD^+^ underwent a 13-fold decrease. Consequently, the overall specificity of the CaIDH H^584^R^595^ mutant was 19-fold greater for NADP^+^ than that for NAD^+^. Thus, the monomeric NAD^+^-specific CaIDH was converted to an NADP^+^-dependent enzyme by two mutations in the coenzyme binding sites.

### Biochemical Characterization

**Figure 6:**
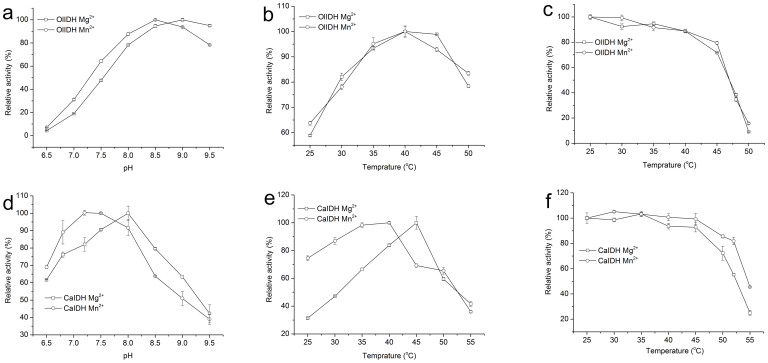
Effects of pH and temperature on the activity of the recombinant OlIDH and CaIDH. (a) and (d), Effects of pH on the activity of OlIDH and CaIDH from pH 6.5 to 9.5 in the presence of Mg^2+^ (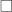) and Mn^2+^ (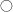), respectively. (b) and (e), Effects of temperature on activity of OlIDH from 25°C to 50°C and CaIDH from 25°C to 55°C in the presence of Mg^2+^ (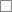) and Mn^2+^ (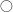), respectively. (c) and (f), Heat-inactivation profiles of the recombinant OlIDH from 25°C to 50°C and CaIDH from 25°C to 55°C in the presence of Mg^2+^ (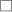) and Mn^2+^ (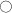), respectively.

The effects of pH on OlIDH and CaIDH activities were determined for the NAD^+^-linked reaction. OlIDH exhibited slightly different pH activity profiles and optimum pH using Mg^2+^ or Mn^2+^ as its cofactor. The optimum pH for OlIDH was pH 9.0 or pH 8.5 in the presence of Mg^2+^ or Mn^2+^, respectively (Fig. 6a). Furthermore, the optimum pH for CaIDH was pH 8.0 or pH 7.5 in the presence of Mg^2+^ or Mn^2+^, respectively (Fig. 6d). OlIDH showed similar pH-activity correlation with the homodimeric NAD^+^-specific *Z. mobilis* IDH (8.5 with Mg^2+^and 8.0 with Mn^2+^) and *Streptococcus suis* IDH (7.0 with Mg^2+^and 8.5 with Mn^2+^) from the type I subfamily^6^,^39^. CaIDH showed a slightly lower optimum pH when compared to the monomeric NADP-IDH from *Streptomyces lividans* TK54 (9.0 with Mg^2+^and 8.5 with Mn^2+^)^22^.

The optimum reaction temperature for OlIDH was around 40°C with either Mg^2+^ or Mn^2+^ as the cofactor (Fig. 6b). Results from heat inactivation studies demonstrate that recombinant OlIDH was stable below 45°C, but its activity rapidly declined as the temperature was raised. Incubation at 45°C for 20 min caused a 28% or 21% loss of activity in the presence of Mg^2+^ or Mn^2+^ (Fig. 6c), respectively, whereas incubation at 50°C caused a 91% or 84% loss of activity in the presence of Mg^2+^ or Mn^2+^, respectively (Fig. 6c). The optimum temperature for CaIDH activity was around 45°C or 40°C in the presence of Mg^2+^ or Mn^2+^, respectively (Fig. 6e). Recombinant CaIDH retained the majority of the activity below 45°C. However, its activity dropped rapidly as the temperature was raised. Incubation at 55°C for 20 min caused a 55% or 75% loss of activity in the presence of Mg^2+^ or Mn^2+^, respectively (Fig. 6f).

The effects of different metal ions on the activities of OlIDH and CaIDH were examined (Table 2). Both enzymes needed the presence of a divalent cation for catalysis, although fractions of activities were observed for OlIDH (5.8%) and CaIDH (7.9%) when no metal ions were added. Mn^2+^ was the most favorable cation for both OlIDH and CaIDH, and its role could be largely replaced by Mg^2+^ (68.6% for OlIDH and 78.3% for CaIDH). Mn^2+^ has also been found to be the preferred cation for other homodimeric NAD-IDHs from *Streptococcus mutans*, *S. suis* and *Z. mobilis*^6^,^39^,^40^. In addition, Mn^2+^ has been determined as the most favored metal ion for monomeric NADP-IDHs^22^,^40^. OlIDH was completely inactivated by either 2 mM Cu^2+^ or Zn^2+^, whereas Co^2+^ and Ni^2+^ caused a 79.5% and 39% loss in activity, respectively. Other metal ions, such as Ca^2+^, Na^+^, and K^+^, slightly affected OlIDH activity in the presence of Mn^2+^. CaIDH was eliminated by 2 mM Zn^2+^and severely down regulated by Co^2+^ (49%), Ca^2+^ (74%), and Cu^2+^ (77%). Monovalent ions (K^+^, Na^+^, Rb^+^, Li^+^) slightly improved CaIDH activity.

## Discussion

In the present study, four novel IDHs, belong to two novel IDH subfamilies, were reported for the first time. Two of them were the NAD^+^-specific homodimeric IDHs from marine alga, *O*. *lucimarinus* (OlIDH) and *Micromonas* sp. RCC299 (MiIDH), and the other two were the NAD^+^-specific monomeric IDH from pathogens *Campylobacter* sp. FOBRC14 (CaIDH) and *Campylobacter curvus* (CcIDH). OlIDH and MiIDH were found to be the first NAD-IDHs in the type II subfamily, as all members of this subfamily were previously thought to be NADP^+^ specific. CaIDH and CuIDH, however, were found to be the first NAD^+^ specific monomeric IDHs. These four IDHs, together with their NAD^+^ specific counterparts, constituted two separate branches on the phylogenetic tree. Most previous studies have proposed that IDH favors NADP^+^ over NAD^+^ as a coenzyme^22^,^25^,^27^,^31^,^34^,^40^. However, as more and more NAD-IDHs from diverse backgrounds being reported, NAD^+^ appears to be widely used by IDH through nature^6^,^26^,^39^,^41^.

By adding two groups of OlIDH-like and CaIDH-like NAD-IDHs, we have expanded and refined the evolutionary classification of the IDH protein family. The phylogenetic tree presented in Fig. 1 contains three well-supported monophyletic groups: type I IDH, type II IDH, and monomeric IDH. Monomeric IDHs were also included in the present phylogenetic analysis for the integrity of IDH family, and the same principle was applied by Delbaere et al.^20^. The most important advancement was the discovery of type II homodimeric NAD-IDHs and monomeric NAD-IDHs, which completed the classification of the IDH protein family in the view of coenzyme specificity. Neither of these groups of NAD-IDH have been reported in previous studies^4^,^23^,^24^,^25^. IDHs with NAD^+^ specificity are ancestral to NADP-IDHs, and this evolutionary hypothesis has been demonstrated by experimental reverse evolution, which was applied to the typical type I *E. coli* NADP-IDH^4^,^23^,^35^. The findings of NAD^+^-specific OlIDH and CaIDH, together with their homologous proteins, will help identify the possible ancestors of type II IDHs and monomeric IDHs, respectively.

By searching the genome of *O*. *lucimarinus* CCE9901 (GenBank Assembly ID: GCA_000092065.1), one copy of *IDH* gene could be found, which suggests that OlIDH is likely the only functional IDH isozyme in this marine algae. The genus *Ostreococcus* is composed of a group of globally distributed, photosynthetic, unicellular green algae, and these cells are the smallest known eukaryotes^42^. Because all known NAD-IDHs from eukaryotes are hetero-oligomeric and consist of at least two different subunits^3^,^43^, OlIDH represents the first eukaryotic homodimeric NAD-IDH. Furthermore, IDHs from some other marine algae, such as *Micromonas* sp. RCC299, *Emiliania huxleyi* and *Thalassiosira oceanica*(see Supplementary Table S1 online), showed high homology to OlIDH (>70%) and branched together with OlIDH on the phylogenetic tree. Considering the antiquity of oceanic algae, the ancient trait of NAD^+^ specificity that was possessed by IDHs in these organisms can be fairly explained.

Similar to OlIDH, the monomeric CaIDH is likely the only active IDH isozyme found in *Campylobacter* sp. FOBRC14 (GenBank Assembly ID: GCA_000287855.1). The monomeric NAD-IDH group seemed to be very small in composition, because only IDHs from the genus *Campylobacter* were included (see Supplementary Table S1 online). These monomeric IDHs shared very high sequence identity (>60%), and the putative NAD^+^-binding sites were conservative (Fig. 2). *Campylobacter* is the most common cause of bacterial foodborne illness and has drawn a lot of attention in recent years^44^,^45^. As a pathogen, it is surprising that the glyoxylate bypass is absent in *Campylobacter* sp. FOBRC14, as no genes encoding isocitrate lyase and malate synthase can be found in its genome. The glyoxylate bypass ensures the bypass of two oxidative steps of the TCA cycle and permits the net incorporation of carbon during the growth of most microorganisms on acetate or fatty acids as the primary carbon source. The end products of the bypass can be used for gluconeogenesis and other biosynthetic processes^46^. Most intracellular human pathogens, such as *Salmonella typhimurium*^47^, *Burkholderia pseudomallei*^48^ and *M. tuberculosis*^49^, need the glyoxylate bypass for their virulence, because fatty acids are the only abundant sources of C2 carbon in mammalian tissues^50^. Interestingly, the existence of the glyoxylate bypass in microorganisms has always been accompanied by at least one NADP-IDH isozyme, which provides the majority of NADPH to support bacterial growth on limited carbon sources^4^. Because the glyoxylate bypass could not be detectedin *Campylobacter* sp. FOBRC14, it is understandable that an NAD^+^-specific IDH, rather than an NADP^+^-specific IDH, was found in this organism. The finding also suggests that the infection mechanism of *Campylobacter* sp. FOBRC14 may be different from that of pathogens with the glyoxylate bypass.

As an eukaryotic NAD-IDH, OlIDH shared very similar kinetic properties with hetero-oligomeric NAD-IDHs from other eukaryotic cells. The *K*_m_ value for NAD^+^ of OlIDH (136.6 μM) was in a similar range of those determined for *Yarrowia lipolytica* yeast (136 μM)^51^ and rats (148.9 μM)^52^. It was higher than that for humans (70 μM)^53^ and lower than that for budding yeast (210 μM)^54^. When compared to homodimeric NADP-IDHs of type II subfamily, OlIDH showed much lower affinity to its coenzyme than NADP-IDHs do to NADP^+^, such as in the wild pig (5.6 μM)^55^, rat (11.5 μM)^56^ and budding yeast (20 μM)^57^. Due to the decrease in cofactor affinity, OlIDH has much lower *k*_cat_/*K*_m_ (0.444 μM^−1^ s^−1^) than its type II NADP-IDH counterparts (5.96 μM^−1^ s^−1^ for wild pig and 9.1 μM^−1^ s^−1^ for rat)^55^,^57^. The poor performance of OlIDH in catalysis may be understood as a latent ancient phenotype, thereby providing more evidence for the age of OlIDH among the type II subfamily. The comparison of kinetic parameters shows that the preference of CaIDH for NAD^+^ over NADP^+^ (61-fold) was significantly lower than that of monomeric NADP-IDHs, such as *S*. *lividans* TK54 (85,000-fold)^22^ and *C*. *glutamicum* (50,000-fold)^27^, thus making CaIDH an old and ineffective enzyme in using NAD^+^. Both OlIDH and CaIDH represent ancient members in the type II and monomeric subfamily, respectively. The modern, sophisticated type II homodimeric NADP-IDH and monomeric NADP-IDH are very possibly refined from old NAD^+^-utilizing ancestors through evolution, as partially evidenced by the fact that just two point mutations in the coenzyme-binding sites of OlIDH and CaIDH were sufficient in converting them to NADP^+^-utilizing enzymes (Table 1).

## Conclusion

In the present study, we refined and expanded the phylogenetic classification of the IDH protein family by dividing the type I, type II, and monomeric subfamilies and identifying two new groups: one group of type II NAD-IDHs, represented by OlIDH, and one monomeric NAD-IDHs, represented by CaIDH. Thus, the classification of the IDH protein family in the view of coenzyme specificity is now complete. OlIDH and CaIDH were heterologously produced and enzymatically characterized in detail. Although the NAD^+^ specificity of the two enzymes was confirmed by kinetic analysis, both enzymes were demonstrated to be ineffective NAD^+^-utilizing enzymes. The coenzyme specificity of both enzymes could be completely altered from NAD^+^ to NADP^+^ by merely mutating two coenzyme-binding amino acids, thus suggesting the ancestral positions of OlIDH and CaIDH in the type II and monomeric subfamilies, respectively. Further studies are clearly needed to understand these two novel groups of IDH in the areas of structure determination and catalytic mechanism investigation.

## Methods

### Gene Synthesis

IDHs from *Ostreococcus lucimarinus* CCE9901 (OlIDH, GenBank accession number: ABP01147), *Micromonas* sp. RCC299 (MiIDH, GenBank accession number: XP_002502450), *Campylobacter* sp. FOBRC14 (CaIDH, GenBank accession number: EJP74315) and *Campylobacter curvus* (CcIDH, GenBank accession number: WP_018136314) were the four targets of this study. Full-length genes encoding these four proteins were synthesized through the gene synthesis service by Generay Biotech Co., Ltd. (Shanghai, China). The coding sequences for four genes were codon optimized by selecting only the most preferential codons according to the *Escherichia coli* bias. The artificial genes were then inserted into the expression vector, pET-28b (+), between the *Nde* I and *Xho* I sites, thus generating four recombinant plasmids, pET-*OIIDH,* pET-*MiIDH,* pET-*CaIDH and* pET-*CcIDH*. The gene sequences were confirmed by sequencing.

### Site-Directed Mutagenesis

Point mutations (Asp326Arg and Met327His) were introduced into OlIDH by overlap extension, PCR-based, site-directed mutagenesis. The oligonucleotides that were used to generate the OlIDH mutant were as follows: forward, 5′-CACGGCACGGCCCACCGTCATTATCTGCGGTATCTC-3′, and reverse, 5′-GAGATACCGCAGATAATGACGGTGGGCCGTGCCGTG-3′. The underlined codons are mutated sequences. The mutated version of CaIDH harboring the Leu584His and Asp594Arg mutations was constructed by two rounds of site-directed mutagenesis. The first round of mutation introduced the Leu584Hischange, using the following oligonucleotides: forward, 5′-GCGGCACTGCTCCGATGCACGCACGCGATATGATCG-3′, and reverse, 5′-CGATCATATCGCGTGCGTGCATCGGAGCAGTGCCGC-3′. The second round of mutation introduced the Asp594Arg change, using the following oligonucleotides: forward, 5′-CGAGCAACCACCTGCGTTGGGATAGCCTGGGCGAG-3′, and reverse, 5′-CTCGCCCAGGCTATCCCAACGCAGGTGGTTGCTCG-3′. The underlined codons are mutated sequences. Mutated genes were then inserted into the expression vector, pET-28b (+), and confirmed by DNA sequencing.

### Overexpression and Purification of Wild-type and Mutated Enzymes

*E. coli* Rosetta (DE3) cells were transformed with pET-*OIIDH,* pET-*MiIDH,* pET-*CaIDH*, pET-*CcIDH*, or recombinant plasmids carrying the mutated IDH genes and grown at 37°C with vigorous shaking in LB medium containing 30 µg/ml kanamycin and 25 µg/ml chloramphenicol. Then, cells were inoculated in 50 ml fresh LB media with the same antibiotic. When the OD_600_ of the culture reached 0.6, isopropyl-1-thio-β-D-galactopyranoside was added to the culture at a final concentration of 0.1 mM with subsequent cultivation overnight at 20°C. Cells were harvested by centrifugation at 4,000 rpm for 15 min and then resuspended in lysis buffer (50 mM Tris-HCl, pH 7.5, 500 mM NaCl). The insoluble debris was removed by centrifugation at 12,000 g for 20 min at 4°C. Then, enzymes with 6×His-tag were purified by using BD TALON Metal Affinity Resins (Clontech, USA), according to the manufacturer's instructions. The expression abundance and purification homogeneity were verified by sodium dodecyl sulfate (SDS)-polyacrylamide gel electrophoresis (PAGE).

### Enzyme Assay

The activities of wild-type and mutant enzymes were assayed by a modification of the method by Cvitkovitch et al.^58^. Activity assays were carried out in 25°C 1-ml cuvettes (1-cm light path) containing 35 mM Tris-HCl buffer (pH 7.5), 2 mM MgCl_2_ or MnCl_2_, 1.5 mM DL-isocitrate, and 1.0 mM NAD^+^. The increase in NADH was monitored at 340 nm with a thermostated Cary 300 UV-Vis spectrophotometer (Varian, USA), using a molar extinction coefficient of 6.22 mM^−1^cm^−1^. One unit of enzyme activity represented the reduction of 1 µM of NAD^+^ per minute. Protein concentrations were determined using the Bio-Rad protein assay kit (Bio-Rad, USA) with bovine serum albumin as the standard.

### Kinetic Analysis

To measure the Michaelis constant (*K*_m_) values of the wild-type and mutant enzymes for NAD^+^ and NADP^+^, the isocitrate concentration was kept fixed at 1.0 mM with varying cofactor concentrations. Apparent maximum velocity (*V*_max_)and *K*_m_ values were calculated by nonlinear regression using Prism 5.0 (Prism, USA). All kinetic parameters were obtained from at least three measurements.

### Temperature and pH Effects

The effects of temperature and pH on the activity of recombinant OlIDH and CaIDH were carried out using the assay method described above. The activities of purified recombinant OlIDH and CaIDH were assayed in 35 mM Tris-HCl buffer between pH 6.5 and 9.5 in the presence of Mn^2+^ (Mg^2+^). The optimum temperature was determined at temperatures that ranged from 25°C–55°C. The thermostability of recombinant OlIDH and CaIDH through heat inactivation were determined by incubating enzyme aliquots at 25°C–50°C for 20 min. After incubation, the aliquots were immediately cooled on ice, and the residual enzyme activity was measured by using the standard enzyme assay.

### Metal Ion Effects

The effects of different metal ions on the activities of recombinant OlIDH and CaIDH were determined using the standard assay method, including 2 mM monovalent ions (K^+^, Li^+^, Na^+^, and Rb^+^) and divalent ions (Ca^2+^, Co^2+^, Cu^2+^, Mg^2+^, Mn^2+^, Ni^2+^, and Zn^2+^).

### Size Exclusion Chromatography (SEC)

The molecular masses of recombinant OlIDH and CaIDH were detected by SEC on a HiLoad^TM^ 10/300 Superdex 200 column (Amersham Biosciences, Germany), which was equilibrated with 0.05 M potassium phosphate buffer (pH 7.0) containing 0.15 M NaCl and 0.01% sodium azide. Protein standards for calibrating gels were ovalbumin (45 kDa), conalbumin (75 kDa), aldolase (158 kDa), ferritin (440 kDa), and thyroglobulin (669 kDa).

### Circular Dichroism Spectroscopy

Circular dichroism (CD) spectroscopy was conducted using a Jasco model J-810 spectropolarimeter (Oklahoma City, OK, USA). The ellipticity measurements, as a function of wavelength, were performed as described previously^59^. Briefly, purified protein samples (0.3 mg/mL) were prepared in 50 mM sodium phosphate and 60 mM NaCl (pH 7.5). The ellipticity (*θ*) was obtained by averaging three scans of the enzyme solution between 200 and 260 nm at 0.5-nm increments. The mean molar ellipticity [*θ*] (deg cm^2^ dmole^−1^) was calculated from [*θ*] = *θ*/10nCl, where *θ* was the measured ellipticity (millidegrees), *C* was the molar concentration of protein, *l* was the cell path length in centimeters (0.1 cm), and *n* was the number of residues per subunit of enzyme (415 for OlIDH and the mutant, 737 for CaIDH and the mutant).

### MALDI-TOF/TOF mass spectrometry

Mass spectrometry analyses were conducted using an AB SCIEX MALDI TOF-TOF 5800 Analyzer (AB SCIEX, USA) equipped with a neodymium: yttrium-aluminum-garnet laser (laser wavelength was 349 nm), in linear high mass positive-ion mode. SA was used as the matrix and TFA was applied as an ionization auxiliary reagent. The TOF/TOF calibration mixtures (AB SCIEX) were used to calibrate the spectrum to a mass tolerance within 10 ppm. The MS spectra were processed using TOF-TOF Series Explorer software (V4.0, AB SCIEX).

### Sequence Alignments and Phylogenetic Analysis

The X-ray structures of *M. tuberculosis* NADP^+^-IDH1 (MtIDH, 4HCX) and *Azotobacter vinelandii* (AvIDH, 1J1W) were downloaded from the Protein Data Bank database (http://www.rcsb.org/pdb/). The homology models of OlIDH and CaIDH were generated by the SWISS-MODEL server (http://swissmodel.expasy.org). The structure-based amino acid sequence alignment was conducted with the Clustal X program (ftp://ftp.ebi.ac.uk/pub/software/clustalw2) and ESPript 3.0 web tool (http://espript.ibcp.fr/ESPript/ESPript/)^60^,^61^.

The typical IDH was used as bait to identify similar IDH sequences in protein database by performing BLAST Link search (http://www.ncbi.nlm.nih.gov/sutils/blink.cgi?mode=query). The redundancy of the query results was eliminated by keeping one IDH sequence for each species, while removing all the other identical IDH sequences for the same species. IDH sequences with relative high homology to the bait sequence were taken for phylogenetic analysis, and their coenzyme binding sites were evaluated by sequence alignment in the first place, in order to confirm their identical coenzyme specificity with the query IDH. Other IDH sequences among the search results were discarded either because their coenzyme usages were different from the bait IDH as predicted by coenzyme binding sites alignment, or because their sequence identities with the query IDH were relatively low, which may suggest their different distribution on the phylogenetic tree. By applying this principle, 197 IDH sequences in total, representing the eleven subgroups encompassed by the three IDH subfamilies, were chosen for phylogenetic analysis. IDH sequences from diverse resources were downloaded from GenBank via the National Center for Biotechnology Information web site (http://www.ncbi.nlm.nih.gov/). The bootstrapped neighbor-joining tree was constructed with the MEGA 6 software (http://www.megasoftware.net/), based on the sequence alignment by Clustal X program (ftp://ftp.ebi.ac.uk/pub/software/clustalw2)^60^,^62^. In order to improve the accuracy of the phylogenetic analysis, some other tree building algorithms were also employed, such as UPGMA method, Maximum Likelihood method and Minimum Evolution method. One outgroup of malate dehydrogenase (MDH) was tried into the phylogenetic study in order to examine whether the IDH tree will be disturbed by adding different proteins.

## Author Contributions

P.W. and C. L. performed the experiments. P.W. and G. Z. planned the project. P. W. and G. Z. wrote the manuscript. All authors reviewed the manuscript.

## Supplementary Material

Supplementary Information Supplementary tables and figures

## Figures and Tables

**Table 1:** The kinetic parameters of the wild-type and mutant enzymes

	NAD^+^			NADP^+^			Specificity	
Enzyme	*K*_m_ (μM)	*k*_cat_(s^−1^)	*k*_cat_/*K*_m_ (A)(μM^−1^ s^−1^)	*K*_m_ (μM)	*k*_cat_ (s^−1^)	*k*_cat_/*K*_m_ (B)(μM^−1^ s^−1^)	(A/B)	(B/A)
OlIDH	136.6 ± 13.6	60.6 ± 3.8	0.444	2211 ± 75	10.0 ± 1.6	0.0045	99	0.009
MiIDH	126 ± 5.3	22.5 ± 2	0.179	1827 ± 42.4	1.4 ± 0.2	0.0008	224	0.004
CaIDH	28.9 ± 4.5	7.0 ± 0.8	0.242	513.2 ± 14.9	1.9 ± 0.1	0.004	61	0.017
CcIDH	74.2 ± 8.2	10.8 ± 1.4	0.146	475.9 ± 6.5	2 ± 0.2	0.004	37	0.027
OlIDH R^326^H^327^	2941.3 ± 75.8	29.1 ± 7.9	0.01	8.2 ± 1.9	1.8 ± 0.2	0.22	0.045	22
CaIDH H^584^R^595^	492.8 ± 52.5	9.5 ± 1.8	0.0193	11.4 ± 4.6	4.2 ± 1.0	0.368	0.052	19

**Table 2:** Effects of metal ions on the activity of the recombinant OlIDH and CaIDH

Metal ions	Relative activity (%) OlIDH	Relative activity (%) CaIDH
None	5.8 ± 1.4	7.9 ± 2.7
Mn^2+^	100.0 ± 3.2	100.0 ± 3.7
Mg^2+^	68.6 ± 0.2	78.3 ± 2.8
Co^2+^	2.8 ± 0.1	35.5 ± 0.4
Ca^2+^	13.6 ± 2.1	0
Zn^2+^	0	0
Cu^2+^	0	0
Ni^2+^	4.1 ± 0.2	8.2 ± 0.9
K^+^	8.7 ± 1.4	1.5 ± 0.1
Na^+^	8.7 ± 0.6	3.6 ± 0.2
Rb^+^	11.0 ± 0.9	2.1 ± 1.8
Li^+^	8.7 ± 0.3	1.3 ± 0.5
Mn^2+^	100 ± 3.2	100 ± 3.7
Mn^2+^+Mg^2+^	90.8 ± 0.8	83.9 ± 2.1
Mn^2+^+Co^2+^	20.5 ± 0.1	50.9 ± 2.4
Mn^2+^+Ca^2+^	89.1 ± 2.9	36.4 ± 0.4
Mn^2+^+Zn^2+^	0	0
Mn^2+^+Cu^2+^	0	22.6 ± 2.6
Mn^2+^+Ni^2+^	61.0 ± 3.5	75.2 ± 3.0
Mn^2+^+K^+^	96.7 ± 4.1	105.9 ± 3.7
Mn^2+^+Na^+^	94.4 ± 0.2	101.4 ± 0.9
Mn^2+^+Rb^+^	88.8 ± 0.5	102.3 ± 1.4
Mn^2+^+Li^+^	91.2 ± 2.6	109.4 ± 1.4
