# Supplementary material for: Novel Type II and Monomeric NAD+ Specific Isocitrate Dehydrogenases: Phylogenetic Affinity, Enzymatic Characterization, and Evolutionary Implication
Source: Sci Rep. 2015 Mar 16;5:9150. doi: 10.1038/srep09150 (PMC4360740; doi:10.1038/srep09150)
Supplement: Supplementary Information — Supplementary tables and figures [file srep09150-s1.pdf]

Novel Type II and Monomeric NAD$^+$ Specific Isocitrate Dehydrogenases: Phylogenetic Affinity, Enzymatic Characterization, and Evolutionary Implication

Peng Wang, Changqi Lv & Guoping Zhu*

Supplementary Table S1
IDH sequences used in the phylogenetic analysis

Organisms	NCBI GI No.	Assignment	Subfamily
Escherichia coli	157077598	Bacterial homodimeric NADP-IDH	I
Shigella flexneri	30040931	Bacterial homodimeric NADP-IDH	I
Citrobacter freundii	363642442	Bacterial homodimeric NADP-IDH	I
Salmonella enterica	326628107	Bacterial homodimeric NADP-IDH	I
Enterobacter cloacae	295057303	Bacterial homodimeric NADP-IDH	I
Klebsiella pneumoniae	323434959	Bacterial homodimeric NADP-IDH	I
Yersinia pestis	108775800	Bacterial homodimeric NADP-IDH	I
Photorhabdus asymbiotica	253780670	Bacterial homodimeric NADP-IDH	I
Pantoea ananatis	291152101	Bacterial homodimeric NADP-IDH	I
Xenorhabdus bovienii	290475824	Bacterial homodimeric NADP-IDH	I
Serratia symbiotica	320029300	Bacterial homodimeric NADP-IDH	I
Erwinia pyrifoliae	283478709	Bacterial homodimeric NADP-IDH	I
Dickeya dadantii	242131546	Bacterial homodimeric NADP-IDH	I
Aeromonas veronii	328804205	Bacterial homodimeric NADP-IDH	I
Grimontia hollisae	262221076	Bacterial homodimeric NADP-IDH	I
Photobacterium profundum	46912770	Bacterial homodimeric NADP-IDH	I
Ferrimonas balearica	307631891	Bacterial homodimeric NADP-IDH	I
Legionella pneumophila	296106397	Bacterial homodimeric NADP-IDH	I
Thiorhodospira sibirica	349789705	Bacterial homodimeric NADP-IDH	I
Ralstonia syzygii	344172186	Bacterial homodimeric NADP-IDH	I
Ralstonia eutropha	73542441	Bacterial homodimeric NADP-IDH	I
Ralstonia solanacearum	299077700	Bacterial homodimeric NADP-IDH	I
Ralstonia solanacearum	206590345	Bacterial homodimeric NADP-IDH	I
Shigella dysenteriae	86516878	Bacterial homodimeric NADP-IDH	I
Cupriavidus taiwanensis	194290616	Bacterial homodimeric NADP-IDH	I
Coxiella burnetii	6560713	Bacterial homodimeric NADP-IDH	I
Thiocapsa marina	343800494	Bacterial homodimeric NADP-IDH	I
Zymomonas mobilis	335932365	Bacterial homodimeric NAD-IDH	I
Alistipes indistinctus	353348998	Bacterial homodimeric NAD-IDH	I
Bacteroides plebeius	198271302	Bacterial homodimeric NAD-IDH	I
Bacteroides coprocola	189430741	Bacterial homodimeric NAD-IDH	I
Parabacteroides merdae	154087236	Bacterial homodimeric NAD-IDH	I
Bacteroides salanitronis	324316830	Bacterial homodimeric NAD-IDH	I
Leishmania major	68129089	Bacterial homodimeric NAD-IDH	I
Leishmania mexicana	322494759	Bacterial homodimeric NAD-IDH	I
Leishmania braziliensis	134065383	Bacterial homodimeric NAD-IDH	I
Brachyspira murdochii	296019527	Bacterial homodimeric NAD-IDH	I
Brachyspira intermedia	343386386	Bacterial homodimeric NAD-IDH	I
Streptococcus mutans	24379145	Bacterial homodimeric NAD-IDH	I
Streptococcus sanguinis	327468982	Bacterial homodimeric NAD-IDH	I

Acidithiobacillus ferrooxidans	198247247	Bacterial homodimeric NAD-IDH	I
Acidithiobacillus thiooxidans	56783614	Bacterial homodimeric NAD-IDH	I
Acidithiobacillus caldus	254970738	Bacterial homodimeric NAD-IDH	I
Thermus thermophilus	499547960	Bacterial homodimeric NADP-IDH	I
Thermus aquaticus	218243848	Bacterial homodimeric NADP-IDH	I
Thermaerobacter subterraneus	493959873	Bacterial homodimeric NADP-IDH	I
Thermomicrobium roseum	501798813	Bacterial homodimeric NADP-IDH	I
Thermomicrobium roseum	221632664	Bacterial homodimeric NADP-IDH	I
Acidiphilium cryptum	146402702	Bacterial homodimeric NADP-IDH	I
Thermus islandicus	551067142	Bacterial homodimeric NADP-IDH	I
Vitis vinifera	225440013	Mitochondrial hetero-oligomeric NAD-IDH	I
Ricinus communis	223541383	Mitochondrial hetero-oligomeric NAD-IDH	I
Glycine max	356548329	Mitochondrial hetero-oligomeric NAD-IDH	I
Nicotiana tabacum	3021506	Mitochondrial hetero-oligomeric NAD-IDH	I
Populus trichocarpa	222867515	Mitochondrial hetero-oligomeric NAD-IDH	I
Medicago truncatula	355492068	Mitochondrial hetero-oligomeric NAD-IDH	I
Populus trichocarpa	222833380	Mitochondrial hetero-oligomeric NAD-IDH	I
Arabidopsis lyrata	297316860	Mitochondrial hetero-oligomeric NAD-IDH	I
Brassica napus	28974494	Mitochondrial hetero-oligomeric NAD-IDH	I
Oryza sativa	113532256	Mitochondrial hetero-oligomeric NAD-IDH	I
Pan troglodytes	332844443	Mitochondrial hetero-oligomeric NAD-IDH	I
Nomascus leucogenys	332252708	Mitochondrial hetero-oligomeric NAD-IDH	I
Rattus norvegicus	149041699	Mitochondrial hetero-oligomeric NAD-IDH	I
Mus musculus	12845252	Mitochondrial hetero-oligomeric NAD-IDH	I
Homo sapiens	5031777	Mitochondrial hetero-oligomeric NAD-IDH	I
Loxodonta africana	344284270	Mitochondrial hetero-oligomeric NAD-IDH	I
Cavia porcellus	348555599	Mitochondrial hetero-oligomeric NAD-IDH	I
Rattus norvegicus	16758446	Mitochondrial hetero-oligomeric NAD-IDH	I
Heterocephalus glaber	351697802	Mitochondrial hetero-oligomeric NAD-IDH	I
Oryctolagus cuniculus	291410721	Mitochondrial hetero-oligomeric NAD-IDH	I
Sus scrofa	335292262	Mitochondrial hetero-oligomeric NAD-IDH	I
Cricetulus griseus	344247643	Mitochondrial hetero-oligomeric NAD-IDH	I
Methylococcus capsulatus	53802809	Bacterial homotetrameic NAD-IDH	I
Methylomicrobium alcaliphilum	357406353	Bacterial homotetrameic NAD-IDH	I
Methylomicrobium album	333597650	Bacterial homotetrameic NAD-IDH	I
Methylobacter tundripaludum	344259777	Bacterial homotetrameic NAD-IDH	I
Aeromonas salmonicida	356689112	Bacterial homotetrameic NAD-IDH	I
Aeromonas caviae	334703580	Bacterial homotetrameic NAD-IDH	I
Oceanimonas sp. GK1	372984893	Bacterial homotetrameic NAD-IDH	I
Pseudoalteromonas tunicata	88820559	Bacterial homotetrameic NAD-IDH	I
Pseudoalteromonas sp. SM9913	315017241	Bacterial homotetrameic NAD-IDH	I
Pseudoalteromonas atlantica	109701783	Bacterial homotetrameic NAD-IDH	I
Shewanella baltica	160874511	Bacterial homotetrameic NAD-IDH	I
Shewanella piezotolerans	212557843	Bacterial homotetrameic NAD-IDH	I
Idiomarina sp. A28L	336282403	Bacterial homotetrameic NAD-IDH	I
Idiomarina loihiensis	56178979	Bacterial homotetrameic NAD-IDH	I
Rheinheimera sp. A13L	335878174	Bacterial homotetrameic NAD-IDH	I
Alishewanella jeotgali	374568889	Bacterial homotetrameic NAD-IDH	I
Ferrimonas balearica	307632714	Bacterial homotetrameic NAD-IDH	I
Shewanella pealeana	157847798	Bacterial homotetrameic NAD-IDH	I
Glaciecola nitratireducens	347947225	Bacterial homotetrameic NAD-IDH	I
Alteromonas macleodii	239997187	Bacterial homotetrameic NAD-IDH	I
Alteromonadales bacterium	119445928	Bacterial homotetrameic NAD-IDH	I

Salinisphaera shabanensis	334888920	Bacterial homotetrameic NAD-IDH	I
Xylella fastidiosa	71164030	Bacterial homotetrameic NAD-IDH	I
Alcanivorax borkumensis	110646268	Bacterial homotetrameic NAD-IDH	I
Ostreococcus lucimarinus	145357157	Eukaryotic (marine algae) homoimeric NAD-IDH	II
Ostreococcus tauri	308810937	Eukaryotic (marine algae) homoimeric NAD-IDH	II
Emiliania huxleyi	551609963	Eukaryotic (marine algae) homoimeric NAD-IDH	II
Aureococcus anophagefferens	323453085	Eukaryotic (marine algae) homoimeric NAD-IDH	II
Phaeodactylum tricornutum	219124414	Eukaryotic (marine algae) homoimeric NAD-IDH	II
Thalassiosira oceanica	397563254	Eukaryotic (marine algae) homoimeric NAD-IDH	II
Thalassiosira pseudonana	223997060	Eukaryotic (marine algae) homoimeric NAD-IDH	II
Guillardia theta	551642293	Eukaryotic (marine algae) homoimeric NAD-IDH	II
Micromonas pusilla	303275390	Eukaryotic (marine algae) homoimeric NAD-IDH	II
Micromonas sp. RCC299	255077633	Eukaryotic (marine algae) homoimeric NAD-IDH	II
Bathycoccus prasinos	612395833	Eukaryotic (marine algae) homoimeric NAD-IDH	II
Thermotoga maritima	490181617	Bacterial homodimeric/homotetrameric NADP-IDH	II
Thermotoga naphthophila	281374132	Bacterial homodimeric/homotetrameric NADP-IDH	II
Dictyoglomus turgidum	217966649	Bacterial homodimeric/homotetrameric NADP-IDH	II
Holophaga foetida	491176902	Bacterial homodimeric/homotetrameric NADP-IDH	II
Carboxydibrachium pacificum	214035411	Bacterial homodimeric/homotetrameric NADP-IDH	II
Leptospira meyeri	463323035	Bacterial homodimeric/homotetrameric NADP-IDH	II
Mycobacterium tuberculosis	660888957	Bacterial homodimeric NADP-IDH	II
Mycobacterium avium	336459578	Bacterial homodimeric NADP-IDH	II
Mycobacterium intracellulare	254820256	Bacterial homodimeric NADP-IDH	II
Mycobacterium colombiense	342131048	Bacterial homodimeric NADP-IDH	II
Mycobacterium parascrofulaceum	295896462	Bacterial homodimeric NADP-IDH	II
Mycobacterium bovis	31794523	Bacterial homodimeric NADP-IDH	II
Mycobacterium marinum	183174534	Bacterial homodimeric NADP-IDH	II
Mycobacterium canettii	340006513	Bacterial homodimeric NADP-IDH	II
Mycobacterium kansasii	240169086	Bacterial homodimeric NADP-IDH	II
Rhodococcus pyridinivorans	363419195	Bacterial homodimeric NADP-IDH	II
Nocardia farcinica	54014398	Bacterial homodimeric NADP-IDH	II
Nocardia cyriacigeorgica	374844727	Bacterial homodimeric NADP-IDH	II
Amycolicicoccus subflavus	333483861	Bacterial homodimeric NADP-IDH	II
Gordonia araii	358245015	Bacterial homodimeric NADP-IDH	II
Cellvibrio gilvus	336103632	Bacterial homodimeric NADP-IDH	II
Dietzia cinnamea	319437576	Bacterial homodimeric NADP-IDH	II
Cellulomonas flavigena	296022180	Bacterial homodimeric NADP-IDH	II
Thermomonospora curvata	268313363	Bacterial homodimeric NADP-IDH	II
Segniliparus rugosus	316253230	Bacterial homodimeric NADP-IDH	II
Actinosynnema mirum	255924768	Bacterial homodimeric NADP-IDH	II
Sus scrofa	255683404	Eukaryotic homodimeric NADP-IDH	II
Bos taurus	73587323	Eukaryotic homodimeric NADP-IDH	II
Pan troglodytes	114658862	Eukaryotic homodimeric NADP-IDH	II
Homo sapiens	47938312	Eukaryotic homodimeric NADP-IDH	II
Pongo abelii	297697439	Eukaryotic homodimeric NADP-IDH	II
Macaca fascicularis	67970962	Eukaryotic homodimeric NADP-IDH	II
Nomascus leucogenys	332238708	Eukaryotic homodimeric NADP-IDH	II
Mus musculus	225579033	Eukaryotic homodimeric NADP-IDH	II
Cricetulus griseus	344257987	Eukaryotic homodimeric NADP-IDH	II
Heterocephalus glaber	351715546	Eukaryotic homodimeric NADP-IDH	II
Zea mays	194697804	Eukaryotic homodimeric NADP-IDH	II
Oryza sativa	20161528	Eukaryotic homodimeric NADP-IDH	II
Brachypodium distachyon	357135759	Eukaryotic homodimeric NADP-IDH	II

Hordeum vulgare	326493350	Eukaryotic homodimeric NADP-IDH	II
Ricinus communis	223532077	Eukaryotic homodimeric NADP-IDH	II
Daucus carota	3811007	Eukaryotic homodimeric NADP-IDH	II
Apium graveolens	2623962	Eukaryotic homodimeric NADP-IDH	II
Prunus persica	15982950	Eukaryotic homodimeric NADP-IDH	II
Elaeis guineensis	192913030	Eukaryotic homodimeric NADP-IDH	II
Elaeis guineensis	374255965	Eukaryotic homodimeric NADP-IDH	II
Campylobacter sp. FOBRC14	497337014	Monomeric NAD-IDH	Monomeric
Campylobacter sp. 10_1_50	496650123	Monomeric NAD-IDH	Monomeric
Campylobacter curvus	516864121	Monomeric NAD-IDH	Monomeric
Campylobacter concisus	544657644	Monomeric NAD-IDH	Monomeric
Campylobacter concisus 13826	157101492	Monomeric NAD-IDH	Monomeric
Campylobacter concisus ATCC 51561	543209960	Monomeric NAD-IDH	Monomeric
Campylobacter concisus UNSW1	543204589	Monomeric NAD-IDH	Monomeric
Campylobacter concisus UNSW2	543212242	Monomeric NAD-IDH	Monomeric
Campylobacter concisus UNSW3	543201738	Monomeric NAD-IDH	Monomeric
Campylobacter concisus UNSWCD	384577620	Monomeric NAD-IDH	Monomeric
Azotobacter vinelandii	226719832	Monomeric NADP-IDH	Monomeric
Corynebacterium glutamicum	499323308	Monomeric NADP-IDH	Monomeric
Burkholderia phytofirmans	187717241	Monomeric NADP-IDH	Monomeric
Pseudomonas brassicacearum	327378561	Monomeric NADP-IDH	Monomeric
Burkholderia multivorans	221174694	Monomeric NADP-IDH	Monomeric
Janthinobacterium	151281179	Monomeric NADP-IDH	Monomeric
Herminiimonas arsenicoxydans	134096614	Monomeric NADP-IDH	Monomeric
Herminiimonas arsenicoxydans	133740517	Monomeric NADP-IDH	Monomeric
Cellvibrio japonicus	192359542	Monomeric NADP-IDH	Monomeric
Xanthomonas axonopodis	346651357	Monomeric NADP-IDH	Monomeric
Achromobacter piechaudii	292816282	Monomeric NADP-IDH	Monomeric
Stenotrophomonas maltophilia	190014021	Monomeric NADP-IDH	Monomeric
Halomonas sp. HAL1	350599098	Monomeric NADP-IDH	Monomeric
Bordetella avium	187478354	Monomeric NADP-IDH	Monomeric
Xylella fastidiosa	71728605	Monomeric NADP-IDH	Monomeric
Rhodanobacter sp. 2APBS1	351684185	Monomeric NADP-IDH	Monomeric
Magnetospirillum magneticum	82944547	Monomeric NADP-IDH	Monomeric
Halomonas elongata	307545653	Monomeric NADP-IDH	Monomeric
Sideroxydans lithotrophicus	291583548	Monomeric NADP-IDH	Monomeric
Geobacter sulfurreducens	39983446	Monomeric NADP-IDH	Monomeric
Alishewanella jeotgali	374570877	Monomeric NADP-IDH	Monomeric
Vibrio cholerae	327483845	Monomeric NADP-IDH	Monomeric
Chlorobium limicola	21396513	Monomeric NADP-IDH	Monomeric
Fluviicola taffensis	327318255	Monomeric NADP-IDH	Monomeric
Rhodomicrobium vannielii	311219856	Monomeric NADP-IDH	Monomeric
Cyclobacterium marinum	342353102	Monomeric NADP-IDH	Monomeric
Anaeromyxobacter dehalogenans	219954679	Monomeric NADP-IDH	Monomeric
Leadbetterella byssophila	311908333	Monomeric NADP-IDH	Monomeric
Shewanella amazonensis	119767694	Monomeric NADP-IDH	Monomeric
Kangiella koreensis	256822324	Monomeric NADP-IDH	Monomeric
Teredinibacter turnerae	254786017	Monomeric NADP-IDH	Monomeric
Glaciecola nitratireducens	348029549	Monomeric NADP-IDH	Monomeric
Burkholderia cenocepacia	124872192	Monomeric NADP-IDH	Monomeric
Desulfurispirillum indicum	316947378	Monomeric NADP-IDH	Monomeric

Supplementary Figure S1

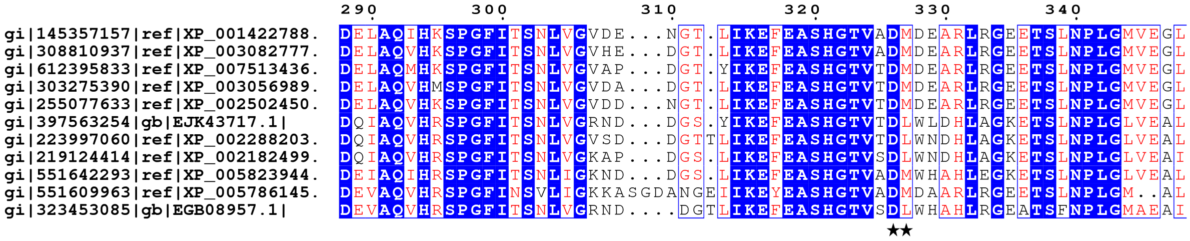

Supplementary Figure S1. Sequence alignment of OlIDH (GI number: 145357157) with other type II NAD-IDHs from marine algae. Protein sequences were represented by their GI numbers. The putative NAD$^+$-binding sites are labeled by ★.

Supplementary Figure S2

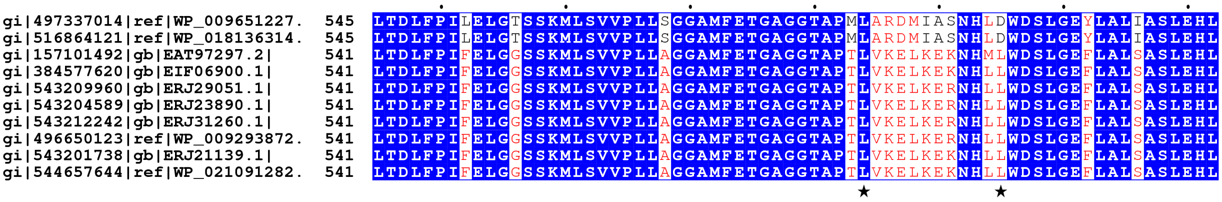

Supplementary Figure S2. Sequence alignment of CaIDH (GI number: 497337014) with other monomeric NAD-IDHs. Protein sequences were represented by their GI numbers. The putative NAD$^+$-binding sites are labeled by ★.

Supplementary Figure S3

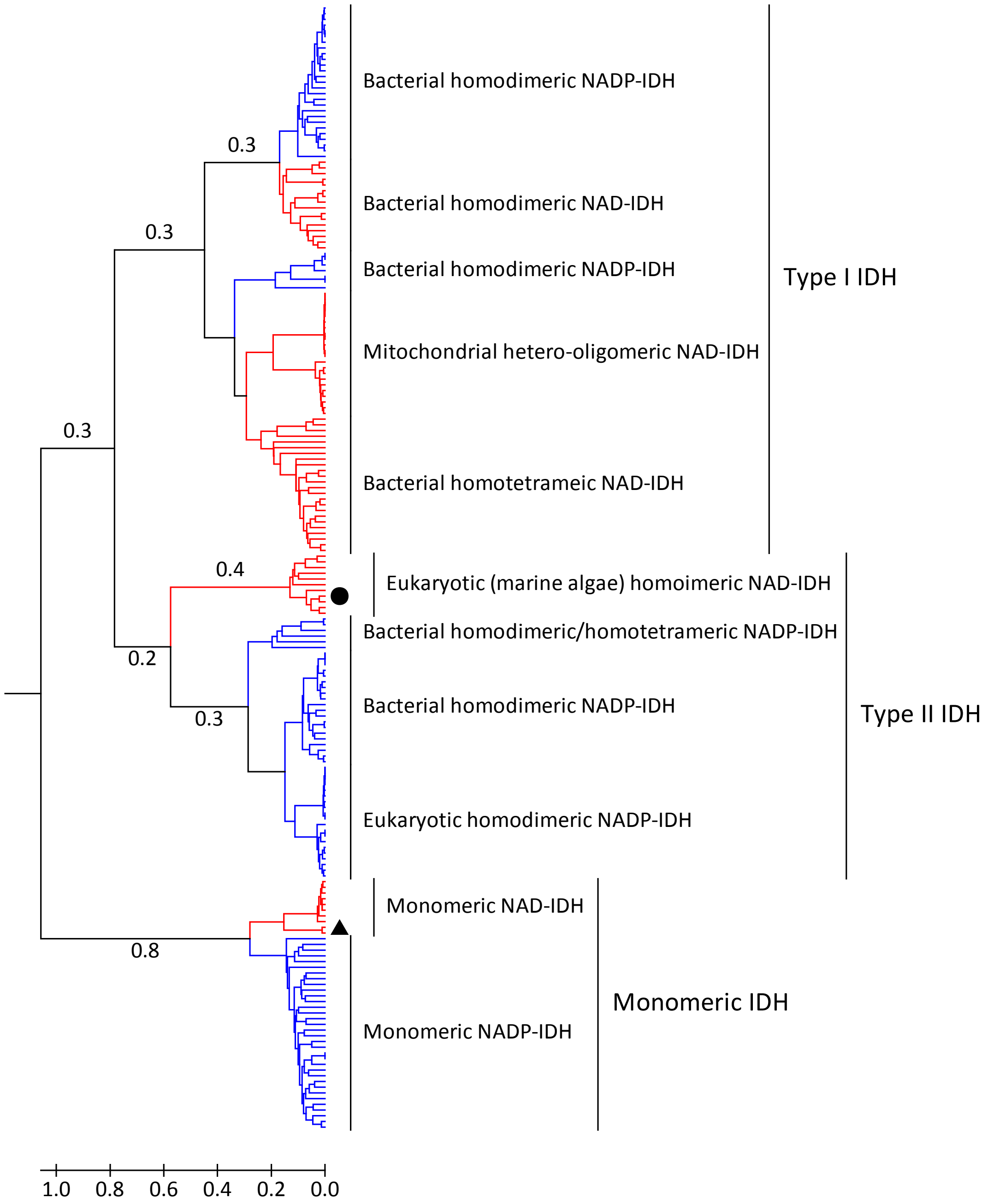

Supplementary Figure S3. Evolutionary relationships of 197 IDHs from diverse background. The evolutionary history was inferred by using the UPGMA method. The optimal tree with the sum of branch length = 15.20995369 is shown. The tree is drawn to scale, with branch lengths in the same units as those of the evolutionary distances used to infer the phylogenetic tree. Phylogenetic analyses were conducted in MEGA6. The IDH sequences used were listed in Table S1. OlIDH and CaIDH were marked by "●" and "▲" respectively.

Supplementary Figure S4

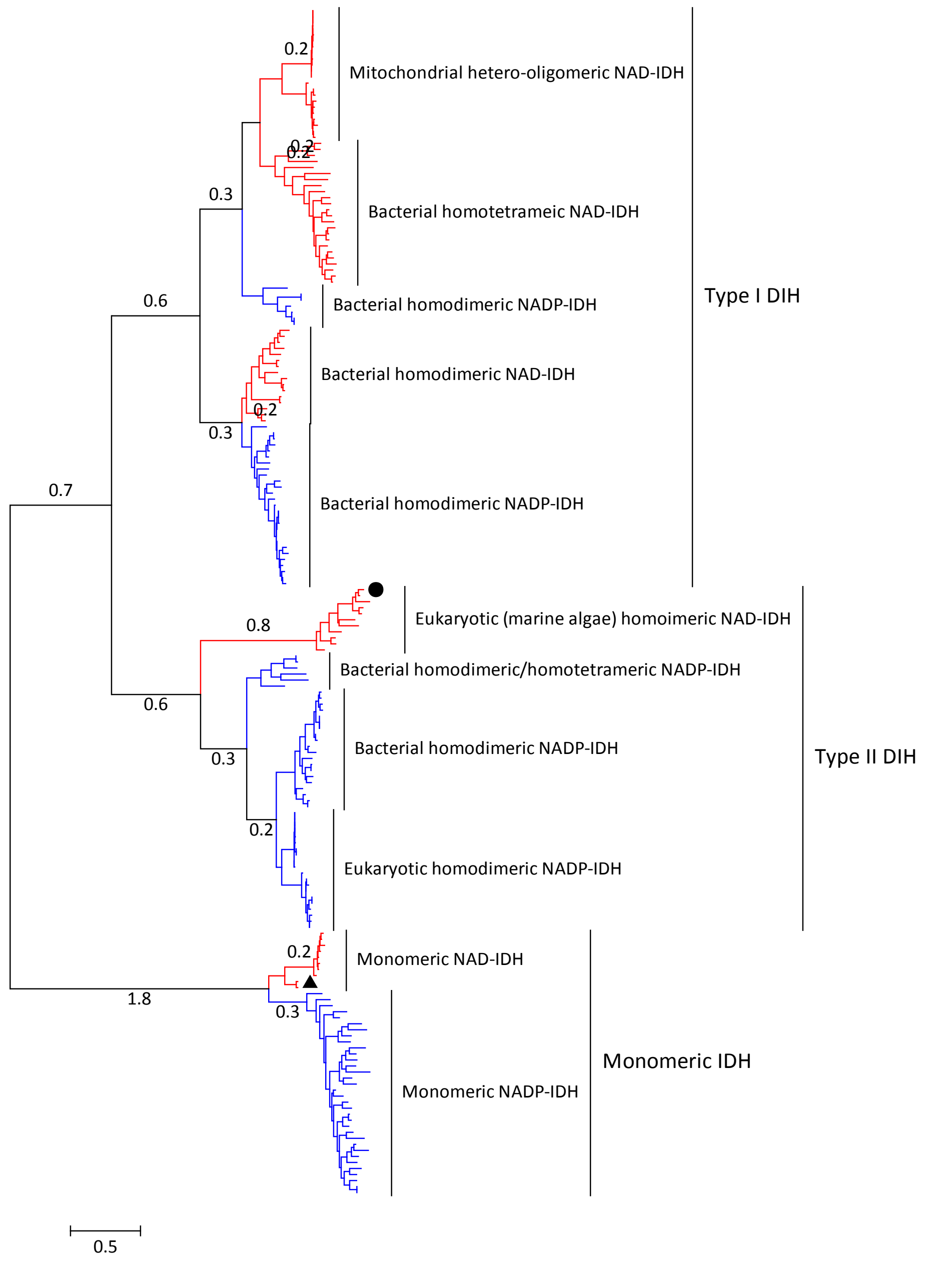

Supplementary Figure S4. Evolutionary relationships of 197 IDHs from diverse background. The evolutionary history was inferred by using the Maximum Likelihood method. Initial tree(s) for the heuristic search were obtained automatically by applying Neighbor-Join and BioNJ algorithms to a matrix of pairwise distances estimated using a JTT model, and then selecting the topology with superior log likelihood value. The tree is drawn to scale, with branch lengths measured in the number of substitutions per site. Phylogenetic analyses were conducted in MEGA6. The IDH sequences used were listed in Table S1. OlIDH and CaIDH were marked by "●" and "▲" respectively.

Supplementary Figure S5

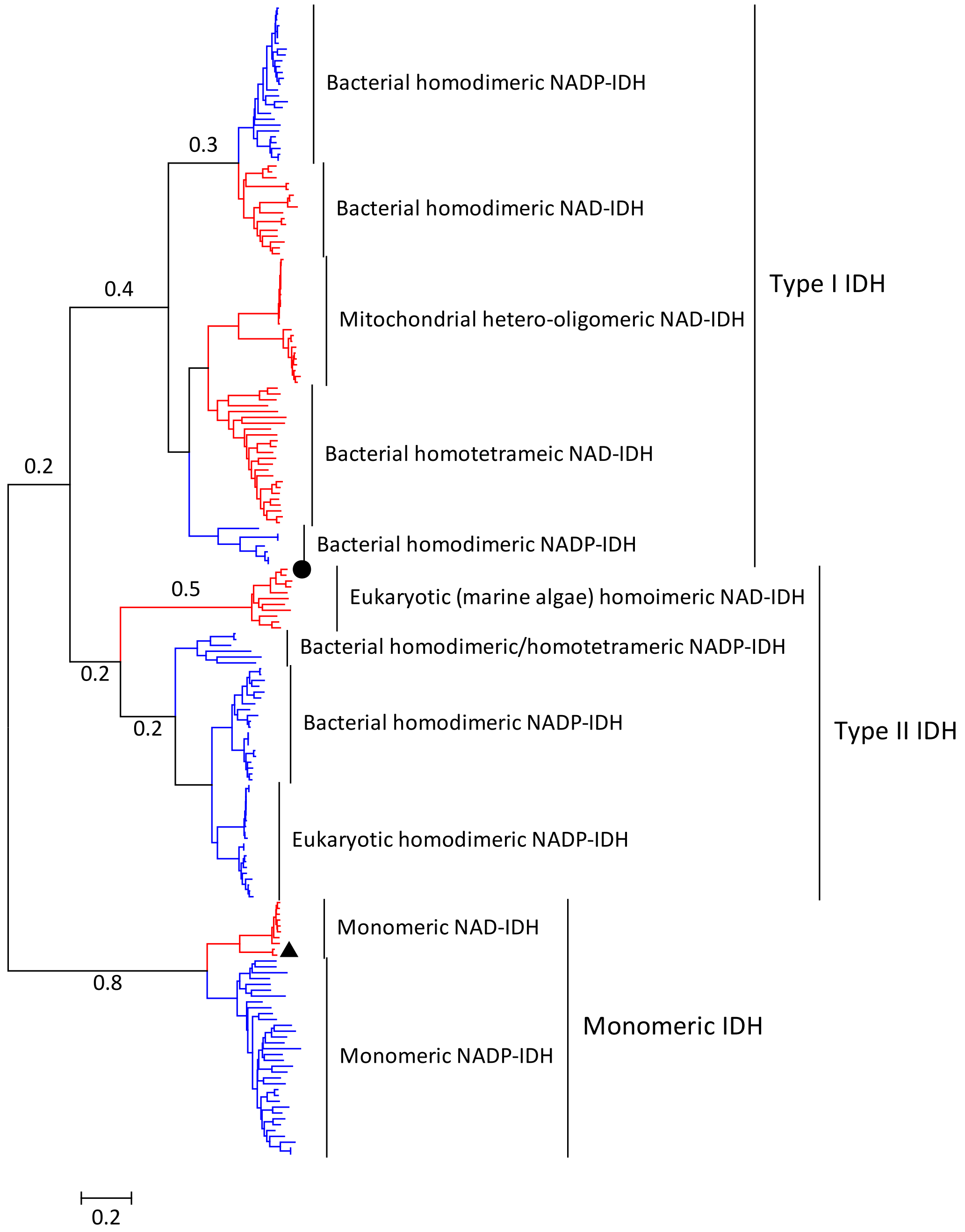

Supplementary Figure S5. Evolutionary relationships of 197 IDHs from diverse background. The evolutionary history was inferred using the Minimum Evolution method. The optimal tree with the sum of branch length = 15.39598692 is shown. The tree is drawn to scale, with branch lengths in the same units as those of the evolutionary distances used to infer the phylogenetic tree. Phylogenetic analyses were conducted in MEGA6. The IDH sequences used were listed in Table S1. OlIDH and CaIDH were marked by "●" and "▲" respectively.

Supplementary Figure S6

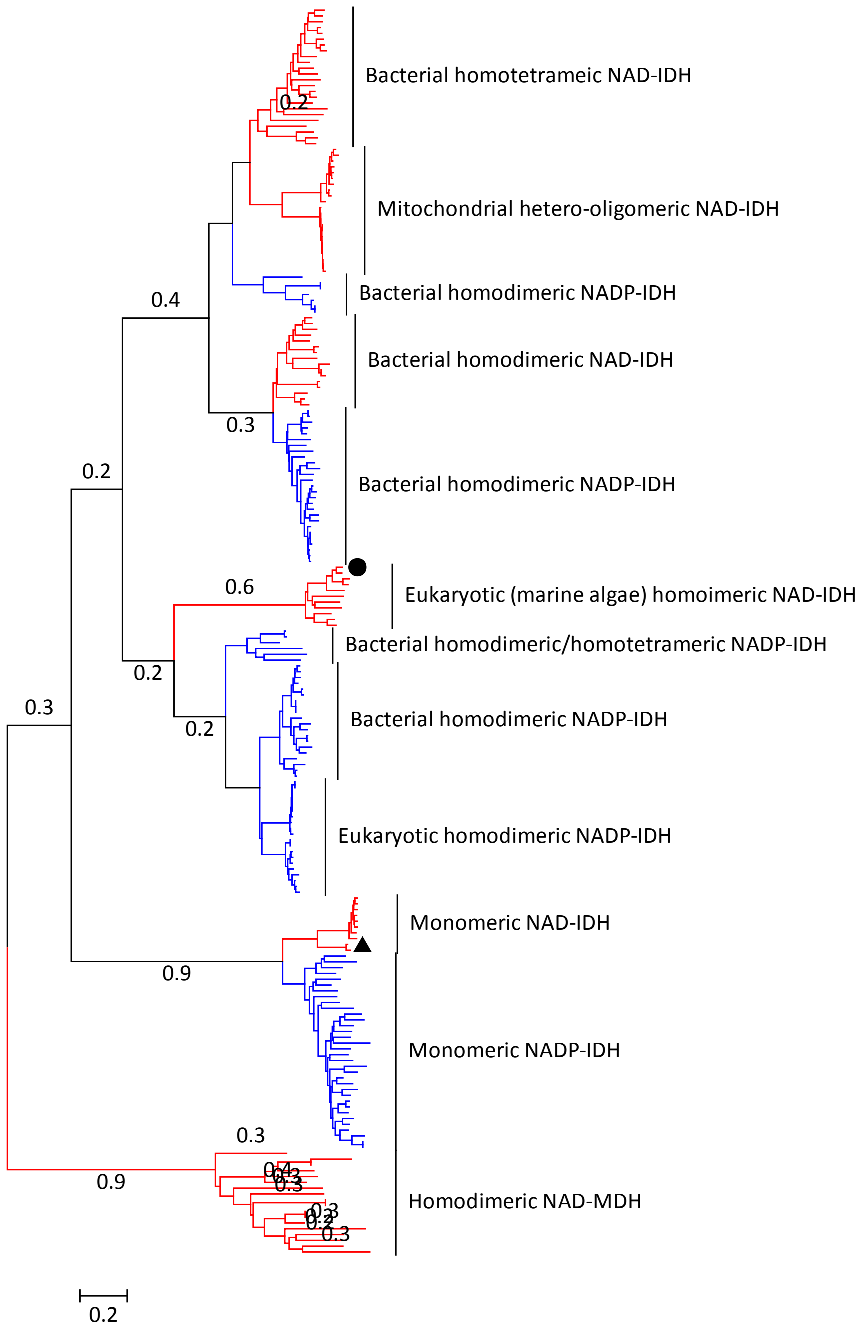

Supplementary Figure S6. Evolutionary relationships of 197 IDHs and 18 MDHs from diverse background. The evolutionary history was inferred using the Neighbor-Joining method. The optimal tree with the sum of branch length = 21.39268747 is shown. The tree is drawn to scale, with branch lengths (next to the branches) in the same units as those of the evolutionary distances used to infer the phylogenetic tree. Phylogenetic analyses were conducted in MEGA6. OlIDH and CaIDH were marked by "●" and "▲" respectively.
